# Important Features for Protein Foldings in Two Acyl Carrier Proteins from *Enterococcus faecalis*

**DOI:** 10.4014/jmb.2309.09006

**Published:** 2023-09-28

**Authors:** Seoyeong Yoo, Jiwon Yeon, Eunhee Kim, Yangmee Kim

**Affiliations:** 1 Department of Bioscience and Biotechnology, Konkuk University, Seoul 05029, Republic of Korea; 2 Center for Research Equipment, Korea Basic Science Institute, Cheongwon-gun, Chungbuk 363-883, Republic of Korea

**Keywords:** *Enterococcus faecalis*, acyl carrier protein, NMR spectroscopy, protein folding, hydrogen/deuterium exchange

## Abstract

The emergence of multi-drug resistant *Enterococcus faecalis* raises a serious threat to global public health. *E. faecalis* is a gram-positive intestinal commensal bacterium found in humans. *E. faecalis* can endure extreme environments such as high temperature, pressure, and high salt, which facilitates them to cause infection in hospitals. *E. faecalis* has two acyl carrier proteins, AcpA (EfAcpA) in de novo fatty acid synthesis (FAS) and AcpB (EfAcpB) which utilizes exogenous fatty acids. Previously, we determined the tertiary structures of these two ACPs and investigated their structure-function relationships. Solution structures revealed that overall folding of these two ACPs is similar to those of other bacterial ACPs. However, circular dichroism (CD) experiments showed that the melting temperature of EfAcpA is 76.3°C and that of EfAcpB is 79.2°C, which are much higher than those of other bacterial ACPs. In this study, to understand the origin of their structural stabilities, we verified the important residues for stable folding of these two ACPs by monitoring thermal and chemical denaturation. Hydrogen/deuterium exchange and chemical denaturation experiments on wild-type and mutant proteins revealed that Ile10 of EfAcpA and Ile14 of EfAcpB mediate compact intramolecular packing and promote high thermostability and stable folding. *E. faecalis* may maximize efficiency of FAS and increase adaptability to the environmental stress by having two thermostable ACPs. This study may provide insight into bacterial adaptability and development of antibiotics against multi-drug-resistant *E. faecalis*.

## Introduction

*Enterococcus faecalis* is an important gram-positive bacterium which causes enterococcal infections in humans and animals [1]. *E. faecalis* inhabits the intestinal tract and also lives in soil and water. *E. faecalis* can survive in extreme environments such as high temperatures, high salt, and antibiotic presence [2, 3]. Fatty acid synthesis (FAS) is essential for all living organisms to produce energy sources for cells as well as building blocks for membranes, cell walls, and protein modification [4-6]. Acyl carrier proteins (ACPs) are essential cofactors for fatty acids biosynthesis [4, 7]. Interestingly, *E. faecalis* has two ACPs, *E. faecalis* AcpA (EfAcpA) and *E. faecalis* AcpB (EfAcpB), with distinct roles and low sequence similarities [8]. EfAcpA is essential for de novo FAS while EfAcpB incorporates exogenous fatty acids [8, 9]. As *E. faecalis* shows resistance to antibiotics, such as vancomycin and linezolid, it is important to further understand the mechanism by which *E. faecalis* is resistant to antibiotics and persists in the external environment for human healthcare [10]. It responds to environmental stress using exogenously provided fatty acids and modifying membrane fatty acid components [11, 12].

In our previous studies, we determined the solution structures of EfAcpA and EfAcpB using NMR spectroscopy [13, 14]. We found that EfAcpB has three helices and one short helix III while EfAcpA has only three helices with a long α2α3 loop without short helix. We also found that EfAcpA and EfAcpB showed higher melting temperatures compared to those of other mesophilic ACPs. Circular dichroism (CD) experiments showed that the melting temperature of EfAcpA is 76.3°C while that of EfAcpB is 79.2°C; the Ala mutation for Ile10 on EfAcpA reduced it dramatically by 29.5°C, implying that highly conserved Ile10 of EfAcpA mediates compact intramolecular packing and promotes high thermostability.

In this study, with the aim of clarifying the important residues for the thermal stability of both EfAcps, the origin of their stability was investigated using thermal denaturation and chemical denaturation experiments on their mutants. Comparison of the structures and protein folding of the two ACPs with divergent sequences and functions provides insight into the distinctive roles of the two ACPs in *E. faecalis* FAS.

## Materials and Methods

### Expression and Purification of EfAcpA and EfAcpB

From *E. faecalis*, *acpA* and *acpB* genes were cloned into pET-28a and pET-21a vectors, respectively (Novagen, USA) and the recombinant vectors were transformed into *E. coli* BL21 (DE3) cells. For NMR experiments, we labeled the proteins and ^15^N-labeled proteins were purified as described previously [13]. Using chelating sepharose HP (GE Healthcare, Sweden) with an imidazole gradient in 20 mM Tris buffer at pH 8.0. In case of EfAcpA, the N-terminal His-tag from EfAcpA was cleaved with 0.3% (w/w) thrombin at 25°C for 16 h and the protein was further purified using HiTrap QFF (GE Healthcare). In case of EfAcpB, protein was purified using HiTrap QFF and Resource Q columns (GE Healthcare).

### Hydrogen/Deuterium Exchange Experiments

All NMR spectroscopy experiments were performed using a Bruker Avance 900 MHz spectrometers at Korean Basic Science Institute, Ochang. EfAcpA, EfAcpB and their mutants were dissolved in 25 mM MES buffer with 5mM CaCl_2_ at pH 6.1. For hydrogen/deuterium (H/D) exchange experiments, ^15^N-labeled samples at 0.5 mM were lyophilized, and 100% D_2_O was added immediately before performing NMR experiments. For 15 h, Heteronuclear single quantum coherence (HSQC) spectra were acquired every 10 min. The logP and ΔG_local_ values of the residues were calculated as previously described [15].

### CD Experiments

Using CD spectrometer, the thermal and chemical denaturation of proteins were measured (J1500 spectropolarimeter, Jasco, Japan). Proteins were dissolved in 25 mM MES with 5 mM CaCl_2_ at pH 6.1. CD spectra were measured from 200 to 250 nm at 0.5 nm intervals. The mean values were plotted as ellipticity θ (10^3^ deg cm^2^/dmol). From the mid-points of the lowest and highest ellipticity from 20°C to 95°C at 222 nm, melting temperatures were calculated.

For chemical denaturation experiments with wild-type proteins, Gdn-HCl from 0 M to 7 M were added to the protein solution. For the chemical denaturation experiments of the mutants, Gdn-HCl from 0 M to 6 M were added to the sample. After 16 h incubation, CD spectra at 25°C were acquired and analyzed as described previously [16].

## Results

### Thermostability of ACPs

**Fig. 1:**
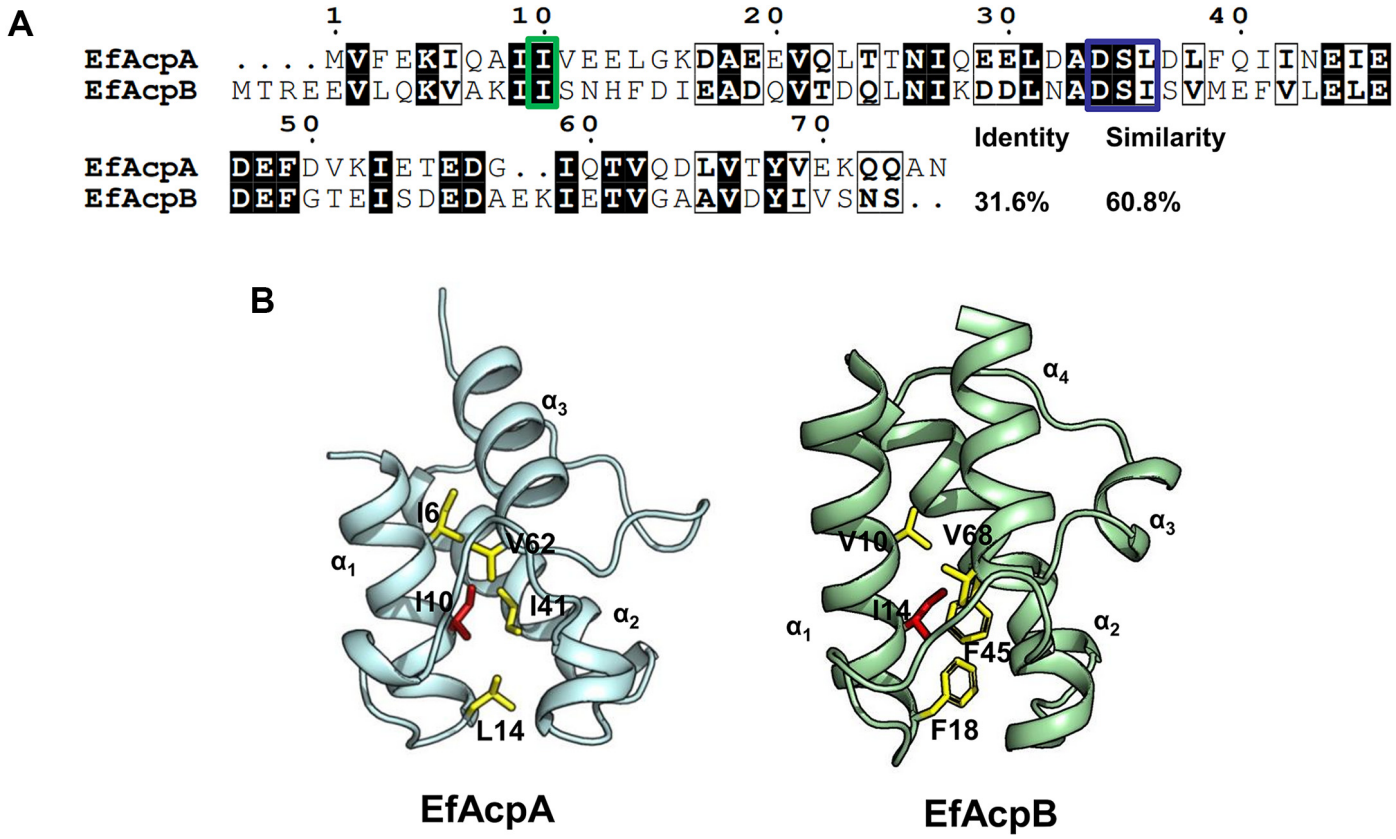
(**A**) Sequence alignment of EfAcpA *and* EfAcpB. ACPs have conserved Ser residues (Ser35 in EfAcpA) near the DSL motif (orange box). Higly conserved Ile, which are important for protein folding, are marked by a green box. (**B**) Solution structure of EfAcpA (PDB ID: 8GSA) and EfAcpB (PDB ID: 2N50) [13, 14]. Conserved Ile residues are marked in red while hydrophobic residues are shown in yellow.

Because *E. faecalis* can tolerate high temperatures, it can be assumed that the ACPs of *E. faecalis* may have thermally stable structures that allow *E. faecalis* to survive and function as an acyl carrier at high temperatures. To investigate the thermostability of EfAcps, we determined the melting temperatures (*T*_m_) of the ACPs using CD experiments. Fig. 1A shows the sequence alignment of the bacterial FAS ACPs, EfAcpA and EfAcpB. Fig. 1A shows that Ile10 and Leu14 in EfAcpA are highly conserved in ACPs involved in de novo fatty acid synthesis. Ile14 in EfAcpB, which corresponds to Ile 10 of EfAcpA is also conserved. In contrast, EfAcpB has Phe18 at the position of Leu14 in EfAcpA. The solution structure of EfAcpB revealed that Phe18 in the α_1_α_2_ loop and Phe45 in helix II of EfAcpB form unique stacking interactions. We previously reported that Ile10 in *Thermotoga maritima* ACP (TmACP) is a critical residue for the thermal stability of TmACP [17], which corresponds to Ile10 in EfAcpA. Fig. 1B shows the hydrophobic interactions between these isoleucine residues and other residue forming the hydrophobic cavity of EfAcpA and EfAcpB, which accommodates growing acyl chains [13, 14].

**Fig. 2:**
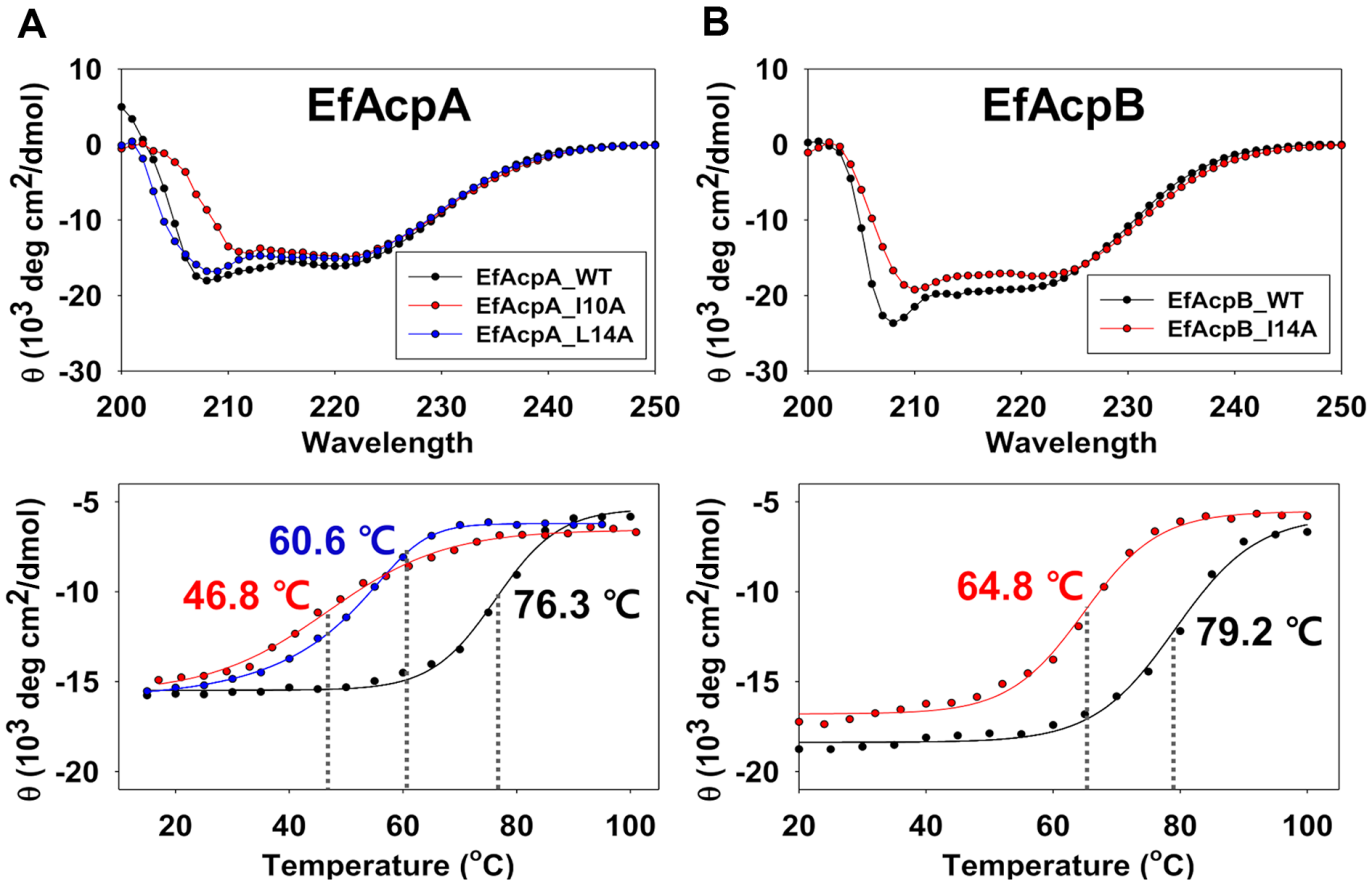
CD spectra of (A) EfAcpA and its mutants, I10A and L14A. (B) EfAcpB and its mutant I14A at 25°C. All samples were in 25 mM MES and 5 mM CaCl_2_ buffer (pH 6.1). At the bottom, temperature-induced folding changes of ACPs were monitored based on changes in ellipticity at 222 nm. *T*_m_ points are marked with a dotted line.

To investigate the effects of specific residues on the thermostability and global folding of EfAcpA, we measured the melting temperatures of the I10A and L14A mutants of EfAcpA (Fig. 2A) as well I14A of EfAcpB (Fig. 2B). *T*_m_ of EfAcpA was 76.3°C while the *T*_m_ of the I10A and L14A mutants of EfAcpA were 46.8 °C and 60.6°C, respectively. Therefore, mutation with replacement of Ile10 by Ala in EfAcpA reduced the *T*_m_ dramatically by 29.5°C while mutation with replacement of Leu14 by Ala in EfAcpA reduced the *T*_m_ also significantly by 15.7°C. These results indicated that Ile10 may be a key residue in the folding process of EfAcpA and Leu14 contributes in a complementary manner. In contrast, I14A mutant of EfAcpB showed decreased thermostability by only 14.4°C, implying that strong hydrophobic interactions between Phe18 and Phe45 of EfAcpB may help to maintain the high thermostability of EfAcpB cooperatively along with Ile14.

### H/D Exchange Experiments

**Fig. 3:**
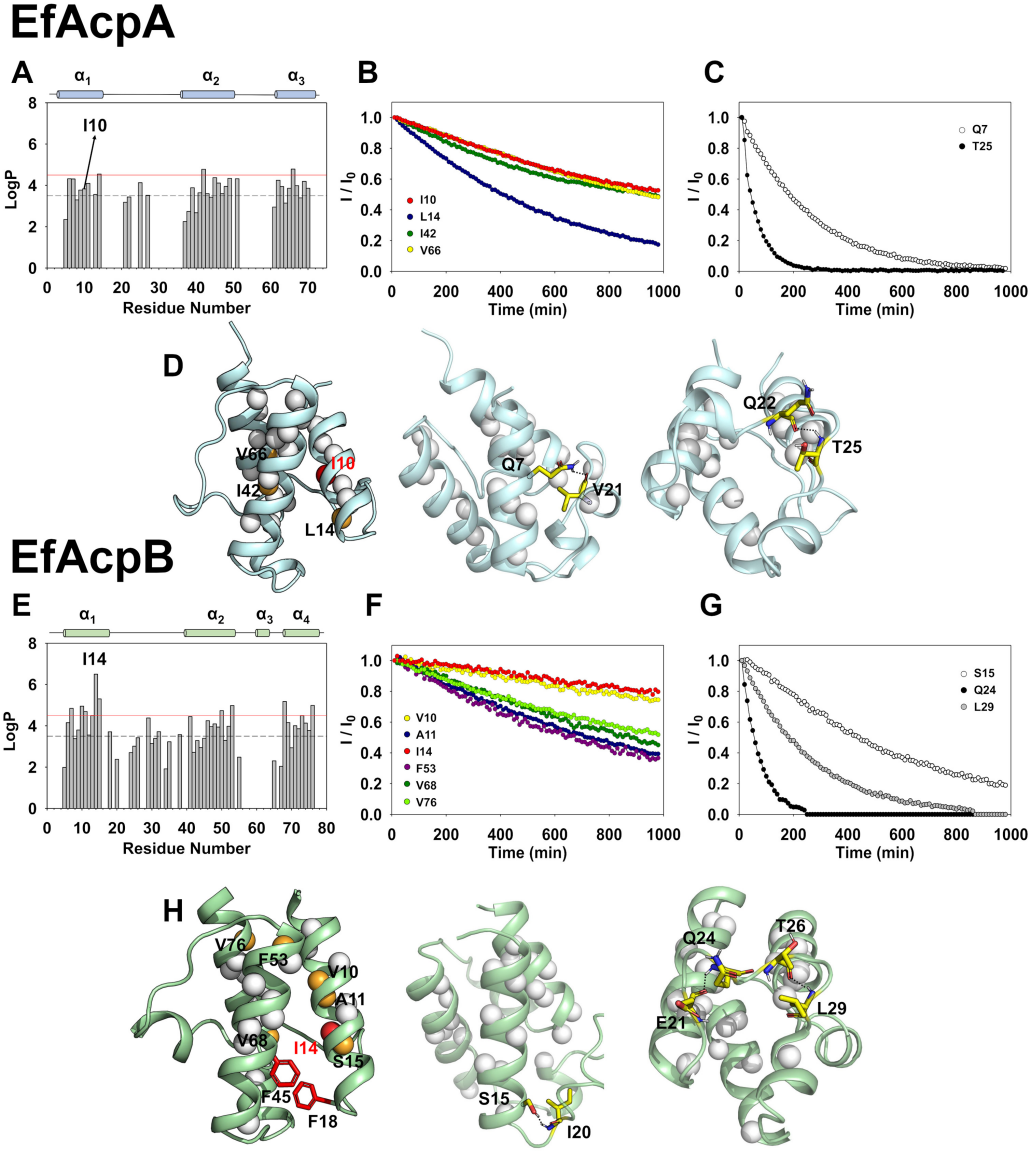
LogP values of amide protons in (A) EfAcpA and (E) EfAcpB from H/D exchange experiments. Residues forming the hydrophobic packing are shown in decay curves of normalized intensities after D_2_O addition (B, F). Residues forming hydrogen bonds (C, G) are indicated in decay curves as a function of time. The amide protons of residues with logP > 3.5 are shown as white spheres and those with logP > 4.5 are shown in orange (D, H). Key residues for the folding of EfAcps are shown by red spheres. Hydrophobic interactions between Phe18 and Phe45 of EfAcpB are shown in red. Residues which form hydrogen bond interactions are depicted as yellow bars. Hydrogen bond interactions are depicted as black dotted lines.

Upon adding D_2_O to protein samples, the amide protons in the protein backbone undergo exchange with deuterium [15]. The H/D exchange rates from the decay curves of the peaks verified the slow exchange rates of the residues, contributing to stable protein structures. H/D exchange experiments were performed for EfAcpA and EfAcpB and the protection factors (logP) of all residues in EfAcpA and EfAcpB were calculated to confirm the protective level of the protein amide proton against H/D exchange. The exchange rate and log values of each residue indicate the regions contributing to the structural stability of ACPs [15, 17]. In general, residues in the helical regions had high logP values. In particular, high protection was observed in the H/D exchange experiment for Ile14 of EfAcpB, which corresponded to Ile10 of EfAcpA (Fig. 3A and 3E). As shown in Fig. 3B and 3F, Ile10 in EfAcpA and Ile14 in EfAcpB marked by red dots had the slowest H/D exchange rates among the important residues involved in folding as well as high protection factors. The exponential decay curves of Ile in both proteins showed low exchange rates, 9.03 × 10^-4^ min^-1^ for Ile10 in EfAcpA, and 2.27 × 10^-5^ min^-1^ for Ile14 in EfAcpB (Fig. 3B and 3F). Furthermore, important residues forming hydrogen bonds to stabilize the α_1_α_2_ loop in EfAcpA and EfAcpB as depicted in Fig. 3C and 3G, show slow exchange, even though they are exposed outward.

Fig. 3D and 3H show the important hydrophobic interactions between these isoleucines and other hydrophobic residues in the cavities as well as the electrostatic interactions in the α_1_α_2_ loops of the two ACPs. Leu14, Ile42, and Val 66 of EfAcpA had high protection factors (> 4.5), implying that those tight hydrophobic interactions between these residues in the cavity resulted in high protection against an exchange. Fig. 3D shows the compact hydrophobic packing via Ile10 in EfAcpA, resulting in slow H/D exchange rates. For EfAcpB, Val10, Ala11, Ile14, Ser15, Phe53, Val68, and Val76 showed high protection factors (> 4.5) and slow decay (Fig. 3E and 3F). As shown in Fig. 3H, the distinctive hydrophobic interactions in red between Phe18 and Phe45 of EfAcpB critical for its thermostability of EfAcpB.

To elucidate the importance of these Ile in the folding of each protein, we next conducted H/D exchange experiments using the mutants and calculated the local unfolding energy (ΔG_local_). The exchanges of EfACPs follow EX2 model for slowly exchanging amide protons [18], ΔG_local_ could be derived from exchange rate constants (K_ex_) and exchange rate of random coil conformation (K_rc_) [18]. Since the K_rc_ value is affected by temperature, solution pH, and local amino acid sequence, we corrected it according to our experimental values [19]. In EX2 model, the K_ex_ is multiplied by the K_unfold_ and K_rc_ values, where K_unfold_ is local unfolding equilibrium constant. Using Eq. (1), we calculated the local unfolding energy (ΔG_local_) [18]

$${\text{ΔG}}_{\text{local}}{\text{=-RT ln (K}}_{\text{unfold}}\text{)}$$ (1)

**Fig. 4:**
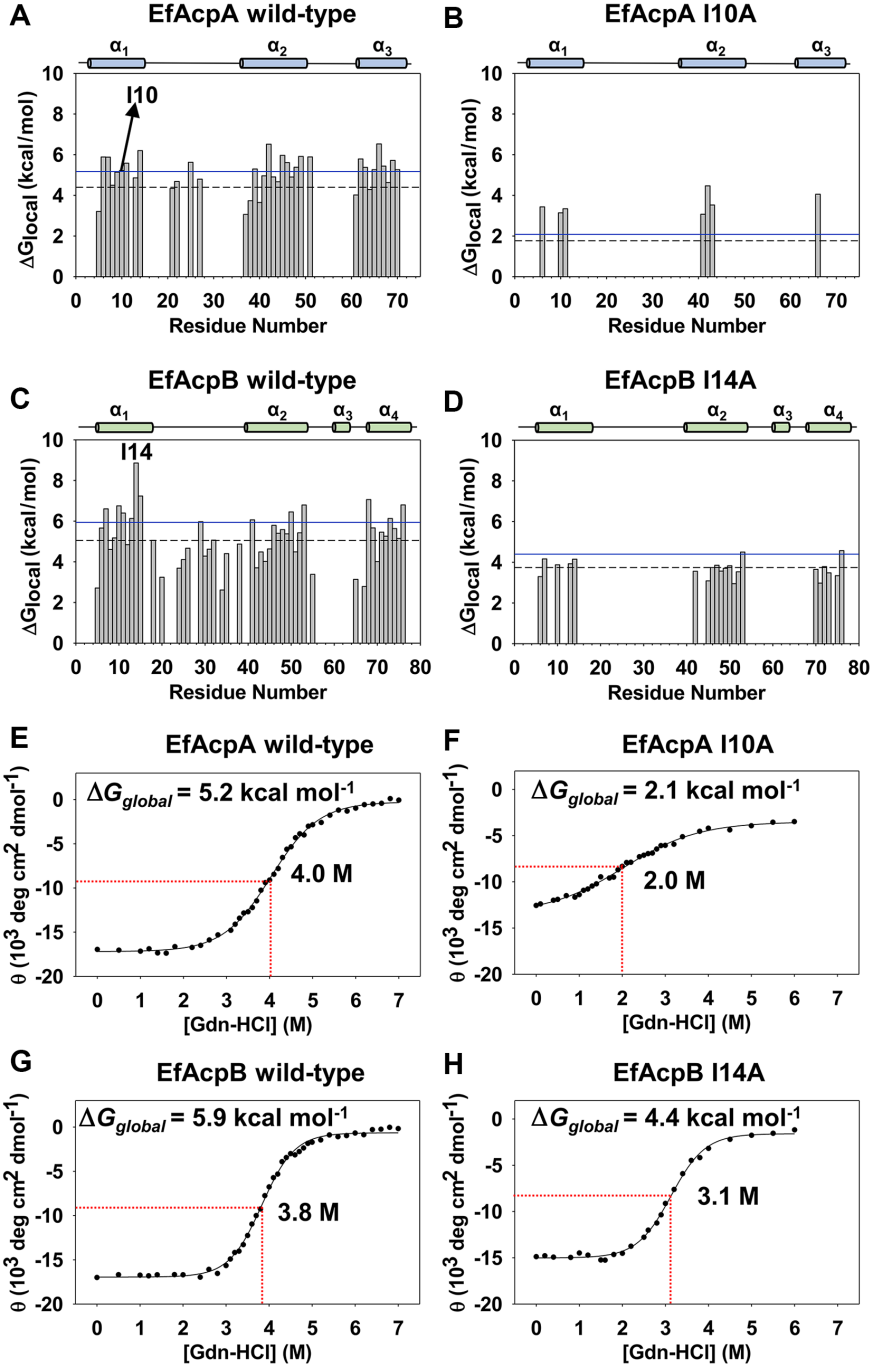
H/D exchange results of (**A**) Wild-type EfAcpA, (**B**) I10A mutant EfAcpA, (**C**)Wild-type EfAcpB, and (**D**) I14A mutant EfAcpB. Blue solid lines in the free energy of local unfolding plots indicate the respective free energy of global unfolding (ΔG_global_) at 25°C. Black dashed lines indicate the lower limit of the global unfolding regime, 0.85ΔG_global_. Gdn-HCl denaturation results of (**E**)Wild-type EfAcpA, (**F**) I10A mutant EfAcpA, (**G**)Wild-type EfAcpB and (**H**) I14A mutant EfAcpB. Red dashed lines in the Gdn-HCl denaturation curves indicate the midpoint concentration [Gdn-HCl]_1/2_.

Where R is the gas constant, and T is 25°C. The I10A mutant of EfAcpA had only seven amide peaks (Ile6, Ala10, Val11, Ile41, Ile42, Gln43, and Val66) remaining after 10 min, which were exchanged much faster than those of the wild-type. These remaining residues were protected owing to hydrophobic interactions in the cavity. All of these disappeared after 60 min. The ΔG_local_ of Ile10 was 5.2 kcal/mol, and its mutation with Ala reduced it by 3.1 kcal/mol (2.1 kcal/mol lower than wild-type) (Fig. 4A and 4B). In addition, the residues forming hydrophobic packing interactions with Ile10 had a lower ΔG_local_ than those in the wild-type protein.

I14A of EfAcpB also displayed fast H/D exchange compared to that of the WT EfAcpB (Fig. 3C). The ΔG_local_ of Ile14 was 8.7 kcal/mol, and its mutation with Ala reduced it by 4.1 kcal/mol (4.6 kcal/mol lower than wild-type)(Fig. 4C and 4D). These results confirm the importance of Ile10 of EfAcpA and Ile14 of EfAcpB for protein folding.

### Chemical Denaturation of Proteins

To understand the role of these important residues in the global folding of each protein, chemical denaturation of proteins using guanidine hydrochloride (Gdn-HCl) was examined [20]. Gdn-HCl induces the global unfolding of proteins, which was observed by monitoring change in the mean residue ellipticity (θ) at a wavelength of 222 nm with different concentrations of Gdn-HCl. The global unfolding energies (ΔG_global_) were calculated using the equation described in previous study [17, 18, 20]. The mid-point concentration ([Gdn-HCl]_1/2_) and ΔG_global_ value of wild-type EfAcpA was 4.0 M and 5.2 kcal/mol, respectively (Fig. 4E). The ΔG_global_ value of the I10A mutant of EfAcpA was much lower (2.1 kcal/mol) than that of the wild-type protein (Fig. 4F).

EfAcpB was also denatured at a similar concentration (3.8 M), but it had a much steeper slope of denaturation, resulting in a much higher ΔG_global_ value (5.9 kcal/mol) than that of EfAcpA (Fig. 4G). These results agree well with those obtained from the melting temperature and H/D exchange experiments. The I14A mutation also destabilized the structure of EfAcpB, resulting in a reduction in the [Gdn-HCl]_1/2_ and ΔG_global_ to 3.1 M and 4.4 kcal/mol, respectively (Fig. 4H). Therefore, we can conclude that Ile10 in helix I of EfAcpA and Ile14 in EfAcpB contribute to the thermostability and global folding of the two ACPs in *E. faecalis*.

## Discussion

*E. faecalis* can endure various environmental stresses, such as acidic, thermal, and oxidative stress during food fermentation and production [21] and has evolved to withstand extreme environments, which might be related to its antibiotic resistance [22]. Because *E. faecalis* also shows high heat tolerance and can survive high temperature up to 85°C [23], we examined the origin of its high heat tolerance by studying its ACPs.

It has been reported that the ACP of the hyperthermophilic protein *T. maritima* (TmACP) has extensive ionic interactions and tight hydrophobic packing, which contribute to its extremely high thermostability (*T*_m_ = 101.4°C)[17]. H/D exchange, chemical denaturation, and mutation studies have shown that Ile15 in TmACP, corresponding to Ile10 in EfAcpA and Ile14 in EfAcpB, is a key residue for its global folding. During H/D exchange experiments, the amide proton of Ile15 in TmACP remained for one month, with a nearly constant peak intensity. The sequence alignment shown in Fig. 1 revealed the importance of Ile, which is well-conserved in bacterial ACPs [17]. Hydrophobic interactions between the nonpolar side chains are known to be important for protein folding. These isoleucines may mediate tight hydrophobic packing in the core of the cavity and are critical for overall ACP folding.

The solution structure and H/D exchange data of EfAcpA demonstrate that additional hydrogen bonding between Gln7 and Val21 as well as Gln22 and Thr25, which stabilize the α_1_α_2_ loop, and hydrophobic packing mediated by Ile10 contribute to the thermostability of EfAcpA. The results of H/D exchange and chemical denaturation experiments confirmed that weakened hydrophobic packing mediated by core Ile residues from Ala substitution reduces the ΔG_local_ and ΔG_global_ of EfAcps. Upon chemical denaturation of mutants, lower ΔG_global_ values were observed, with a decrease of 3.1 kcal/mol for I10A of EfAcpA and 1.5 kcal/mol for I14A of EfAcpB. Although the amide proton of I14 in EfAcpB was highly protected, I14A mutant had less impact on EfAcpB folding as shown in the thermal and chemical denaturation experiments. In our previous study, the melting temperatures of the F18A and F45A mutants of EfAcpB were decreased by 9.3°C, and 25.9°C, respectively [13]. Therefore, the stable hydrophobic interactions formed by Phe18 and Phe45, along with the intramolecular hydrophobic packing mediated by Ile14 are likely to cooperatively stabilize the folding of EfAcpB. These findings on the protein folding and stability of two EfAcps may help us understand how *E. faecalis* can tolerate extreme environments, especially at high temperatures, and how *E. faecalis* resists to antibiotics and persists in the external environment.
